# The Elderly and Waterborne *Cryptosporidium* Infection: Gastroenteritis Hospitalizations before and during the 1993 Milwaukee Outbreak

**DOI:** 10.3201/eid0904.020260

**Published:** 2003-04

**Authors:** Elena N. Naumova, Andrey I. Egorov, Robert D. Morris, Jeffrey K. Griffiths

**Affiliations:** * Tufts University School of Medicine, Boston, Massachusetts, USA

**Keywords:** elderly, cryptosporidiosis, waterborne infection, outbreak, surveillance, susceptible population, time-series analysis, research

## Abstract

We used the Temporal Exposure Response Surfaces modeling technique to examine the association between gastroenteritis-related emergency room visits and hospitalizations in the elderly and drinking water turbidity before and during the 1993 Milwaukee waterborne *Cryptosporidium* outbreak. Before the outbreak, the rate of such events increased with age in the elderly (p<0.002), suggesting that the elderly are at an increased risk. During the outbreak, strong associations between turbidity and gastroenteritis-related emergency room visits and hospitalizations occurred at temporal lags of 5–6 days (consistent with the *Cryptosporidium* incubation period). A pronounced second wave of these illnesses in the elderly peaked at 13 days. This wave represented approximately 40% of all excess cases in the elderly. Our findings suggest that the elderly had an increased risk of severe disease due to *Cryptosporidium* infection, with a shorter incubation period than has been previously reported in all adults and with a high risk for secondary person-to-person transmission.

The elderly are a population at higher risk for infections (*1*). Changes in immune system and gastrointestinal functions occur with aging, leading to increased susceptibility to enteric infections (*2*–*4*). The elderly, along with children and pregnant women, are recognized by the U.S. Environmental Protection Agency as being sensitive subpopulations for waterborne diseases (*5*). Other researchers have recognized the elderly population as a potential sentinel group for surveillance of cryptosporidiosis (*6*). However, the degree of this increased sensitivity to specific gastrointestinal infections is not well characterized. In the United States, most prospective studies of enteric disease in the elderly were conducted two decades ago or earlier, when diagnostic techniques were limited, and many pathogens such as *Cryptosporidium, Cyclospora,* microsporidia, and *Escherichia coli* 0157:H7 were not widely recognized and routinely diagnosed (*7*–*9*). Although diagnostic techniques have improved, a substantial proportion of gastrointestinal illness in the elderly and general population remains routinely undiagnosed. For example, a recent prospective study of gastroenteritis in sentinel general practices in the Netherlands found that the causative agent could be detected in only 40% of all patients (*10*).

Contaminated drinking water is a well-documented route of transmission for *Cryptosporidium parvum* (*11*). Disinfecting water by using chlorination does not inactivate this parasite, making water filtration essential in protecting public water supplies. In the spring of 1993, Milwaukee had an outbreak of waterborne cryptosporidiosis associated with increased contamination of source water and a breakdown in the water filtration process at the Howard Avenue Water Treatment Plant (the south Plant) (*12*), causing a sharp increase in finished water turbidity. More than 400,000 persons became ill, and >100 immunocompromised persons died as a result of *Cryptosporidium* infection. This epidemic was the largest of waterborne disease reported in the United States.

In our previous studies, we demonstrated that the increased rates of acute gastrointestinal illness (gastroenteritis) in Milwaukee were significantly associated with increased drinking water turbidity (13–15). During the outbreak period, the association between drinking water turbidity and physician-diagnosed gastroenteritis was the strongest at a time lag of 7 days in children and 8–9 days in adults (*15*). These time lags correspond to typical incubation periods for *Cryptosporidium*. While experimental animal data demonstrate that the incubation period of cryptosporidiosis is related to immune status and dose of the pathogen (*16*–*18*), little direct information for sensitive human subpopulations exists except for persons with AIDS and malnourished children (*19*).

Our goal was to identify the specific features of the epidemic response in the elderly during the Milwaukee outbreak of cryptosporidiosis in 1993. We sought to identify 1) the magnitude of the association between increased drinking water turbidity and increased rate of gastroenteritis; 2) the median lag between exposure and illness, corresponding to the incubation period; and 3) the magnitude of the secondary spread. We hypothesized that the Milwaukee elderly might be more susceptible to *Cryptosporidium* infection than the nonelderly population. This higher susceptibility could, in principle, be reflected by a shorter time lag for the elderly during the epidemic, a higher overall increase in the rate of gastroenteritis associated with increased turbidity, and an association between the rate of gastroenteritis and older age. In addition, a pronounced second postexposure peak in infections could represent a higher risk for person-to-person transmission among the elderly. Since many elderly live together in nursing homes, the risk for secondary transmission may be increased as well. We used the Health Care Financing Administration (HCFA) database and novel analytical techniques to investigate the temporal, spatial, and demographic patterns in hospitalizations and emergency room visits for acute gastrointestinal illness (GIH events) among the elderly.

## Data and Methods

### Hospitalization and Emergency Room Visits

We extracted all available records of GIH events in persons who were >65 years of age and resided in Milwaukee County, Wisconsin, from the HCFA database for the 480-day period from January 1, 1992, through April 24, 1993. The dataset included age, zip code, type of admission, and International Classification of Disease (ICD)-9 code. We abstracted data on ICD codes 007 through 009, 558.9, and 787.0. These codes include most cases of acute gastrointestinal illness reflected in the HCFA database.

To estimate the daily rate of reported cases of acute gastroenteritis per 100,000 elderly persons, we abstracted data from the 1990 Census for five age groups (65–69, 70–74, 75–79, 80–84, and >85 years of age) for each zip code in Milwaukee County. To estimate the endemic and epidemic daily rate of GIH events, we divided the study time into two parts: pre-outbreak (452 days; January 1, 1992–March 27, 1993) and outbreak (28 days; March 28, 1992–April 24, 1993).

We divided Milwaukee zip codes into three categories according to the drinking water source: the north area (the Linnwood Water Treatment Plant), the south area (the Howard Avenue Water Treatment Plant), and the central area, where water from these two plants is mixed. We estimated mean daily rate of GIH events in each area before and during the outbreak and examined geographic distribution of daily rate of gastroenteritis by using the ARC/View 3.2 GIS software (ESRI, Redlands, CA).

### Association between GIH Infections and Drinking Water Turbidity

As a surrogate measure of exposure to *Cryptosporidium* oocysts, we used daily maximum effluent water turbidity (provided by the Milwaukee Water Works) at the south water treatment plant. To examine temporal associations between effluent water turbidity and gastrointestinal illness, we generated a time series of daily counts of gastroenteritis in the south and central areas, which was regressed to a time series of turbidity data. We performed the analysis by using a Generalized Additive Model (GAM) with a nonparametric “loess” smoother for the exposure variable and a set of linear autoregressive components. The number of autoregressive components was selected by using the bias-corrected Akaike Information Criterion (*13*,*14*). To cover the range of possible incubation periods of cryptosporidiosis (*19*), we conducted this analysis for time lags between exposure and illness from 0 to 18 days, one lag at a time.

To test for the significance of regression slopes, reflecting the relationship between turbidity and daily rate of GIH events at the lags consistent with the incubation period for cryptosporidiosis, we repeated this analysis with the Generalized Log-linear Models (GLM). Although we expected to see the strongest association of lag at 5–9 days, we did not force the model to follow the lag structure based on a theoretical distribution of incubation periods in the population but allowed an equal probability for any lag to be influential on the outcome. Model diagnostics and significance of regression slopes for correspondent lags were tested within the GLM framework. Lags with statistically significant slope estimates for turbidity were identified.

To visualize the lagged relationship between exposure and gastrointestinal illness, we produced the temporal exposure response surface (TERS), which reflected the changes in lagged daily rates of gastroenteritis associated with changes in turbidity. Instead of plotting 18 dose-response curves (one for each lag), we assembled them in a three-dimensional surface aligned by turbidity values. The lags at which both the GLM and GAM models predicted the strongest impact of increased turbidity on the rate of GIH events after adjusting for time-varying covariates were marked on the TERS plot.

For each time lag from 0 to 18 days, we estimated the excess daily rate of gastroenteritis associated with four levels of turbidity: 0.0–0.29, 0.3–0.49, 0.5–0.99, and 1.0–2.0 Nephelometric Turbidity Units (NTU). For a given lag, the excess rate estimate reflects the difference between the predicted epidemic daily rate at a given level of turbidity and the disease-endemic daily rate during the pre-outbreak period. All analysis was performed with S-plus 4.5 statistical software (Insightful Inc., Seattle, WA).

## Results

Daily rates of GIH events per 100,000 elderly persons by age category are listed in Table 1. During and before the outbreak, the age-specific rates of GIH events exhibited similar positive trends; on average, daily rates increased by 0.44 GIH events per 100,000 persons for every 10 additional years of age (p=0.001). During the outbreak, the daily rate was substantially higher in every age category (paired t test, p=0.002) than during the pre-outbreak period.

**Table 1:** Daily rates of gastroenteritis-related emergency room visits and hospitalizations per 100,000 elderly persons by age category before and during the 1993 outbreak of cryptosporidiosis, Milwaukee, Wisconsin

Age group (y)		Before outbreak (452 d)		During outbreak (28 d)	
	Elderly population	No. cases	Daily rate/ 100,000	No. cases	Daily rate/ 100,000
65–69	39,561	66	0.37	15	1.35
70–74	33,019	108	0.72	17	1.84
75–79	26,188	97	0.82	12	1.64
80–84	17,584	93	1.17	16	3.25
85+	14,150	81	1.27	9	2.27
All	130,502	445	0.75	69	1.89

**Figure 1:**
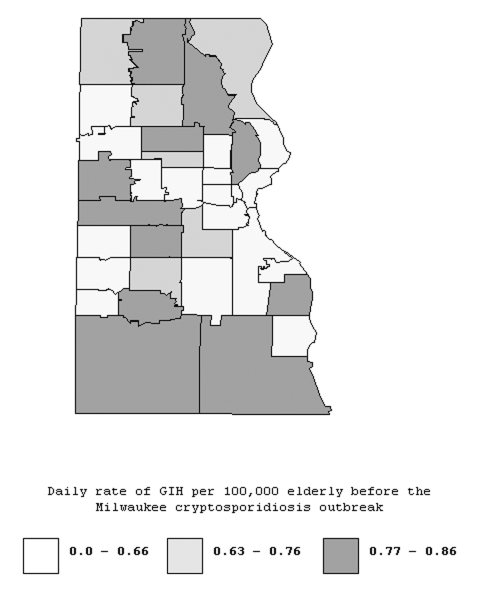
Geographic distributions of age-adjusted daily rate of gastroenteritis-related emergency room visits and hospitalizations per 100,000 elderly persons for the pre-outbreak period (January 1, 1992–March 27, 1993), Milwaukee, Wisconsin.

**Figure 2:**
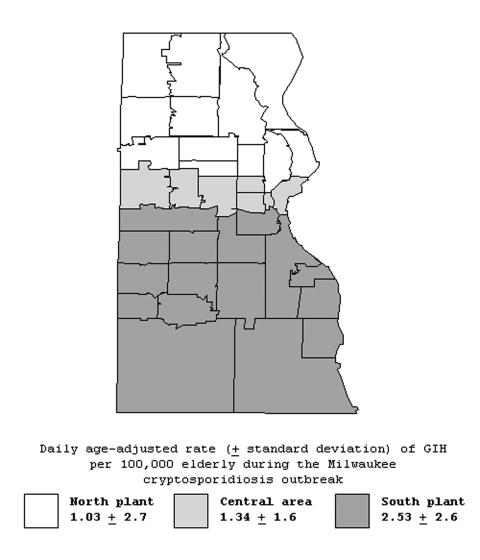
Age-adjusted daily rates of gastroenteritis-related emergency room visits and hospitalizations per 100,000 elderly persons during the cryptosporidiosis outbreak (March 28, 1993–April 24, 1993) in three drinking water service areas (north, central, and south), Milwaukee, Wisconsin.

The geographic distribution GIH events rates for the pre-outbreak period by zip code are shown in Figure 1. This spatial distribution of rates does not suggest any consistent spatial pattern. Before the outbreak, rates of GIH events in the elderly were similar in south, central, and north areas (Table 2). During the outbreak, rates of GIH events in elderly persons increased in all three water supply areas, but the increase was much stronger in the southern and central areas than in the northern area (Figure 2 and Table 2). The daily rate of GIH events in the elderly residing in the southern area during the outbreak was 2.6 times higher than in the northern area.

**Table 2:** Daily rates of gastroenteritis-related emergency room visits and hospitalizations in the elderly by water supply area before and during the 1993 outbreak of cryptosporidiosis, Milwaukee, Wisconsin

			Before outbreak		During outbreak	
Water supply area	No. zip codes	No. elderly	No. cases	Daily rate/ 100,000	No. cases	Daily rate/ 100,000
North	13	50,747	182	0.79	14	0.99
Central	6	18,533	50	0.60	11	2.12
South	15	61,165	213	0.77	44	2.57

**Figure 3:**
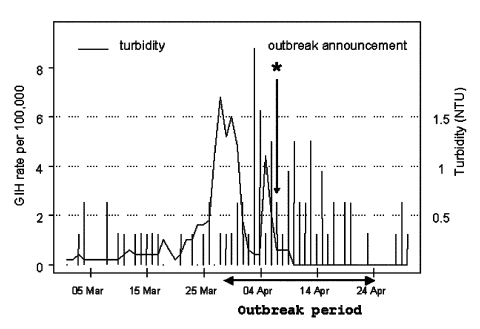
The fragment of the time series of daily rates of gastroenteritis-related emergency room visits and hospitalizations among Milwaukee elderly in the south and central water supply areas and daily water turbidity at the south treatment plant. The outbreak period (March 28, 1993–April 24, 1993) is indicated by blue lines; the day of announcement of the outbreak by the Milwaukee Health Department (April 7, 1993) is indicated by a green star.

The time-series analysis employed turbidity data from the south plant and GIH events data from the south and central areas, which were supplied completely or partially by this plant. The mean (± standard deviation) daily rates of GIH events in these areas before and during the outbreak were 0.74 (±0.94) and 2.48 (±2.07), respectively. Before the outbreak, the daily rate did not exceed 2.5 cases per day in 98% of days. However, during the outbreak, in 7 of 28 days, the daily rate was >2.5 cases per day. Before March 1993, daily turbidity never exceeded 0.25 NTU. Time series of daily rates of GIH events in the south and central areas and daily maximum effluent turbidity for a 60-day period, including the outbreak, are shown in Figure 3.

**Figure 4:**
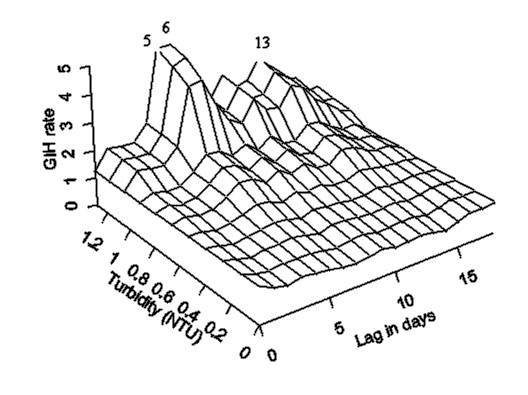
The temporal exposure response surface plot of the lagged association between daily rate of gastroenteritis-related emergency room visits and hospitalizations in the elderly in south and central water supply areas of Milwaukee, Wisconsin (per 100,000) and water turbidity (Nephelometric Turbidity Units) at the south plant.

For the pre-outbreak period, we have not found any statistically significant associations between elevated water turbidity and rates of GIH events at any time lag. During the outbreak, statistically significant associations between elevated water turbidity and rates of GIH events were detected at time lags of 5, 6, 7, and 13 days by both the GLM and GAM models. As expected, no association existed between the exposure and the outcome (GIH events) on the same day at a zero time lag. Associations at other lags from 1 to 18 days were positive but not statistically significant (Figure 4). Based on GLM analysis, the 95% confidence interval for the relative risk associated with 1 NTU increase in turbidity at time lags of 5 and 6 days was 1.54 to 4.48.

The results of modeling of the temporal relationship between turbidity and GIH events in the elderly during the outbreak period are demonstrated by the TERS surface on Figure 4. The strongest association between increased water turbidity and increased rates of GIH events was observed at a lag of 6 days, and the second highest peak was at a lag of 13 days. This second peak is temporally consistent with secondary person-to-person transmission. The flat portion of the surface reflects the absence of any associations at low levels of turbidity (<0.5 NTU) at any lag.

The estimates of excess daily rate of GIH events in the elderly (the difference between the GAM-predicted daily rate during the outbreak and the pre-outbreak daily rate of 0.75 cases/100,000 persons) associated with four levels of turbidity at time lags from 0 to 18 days are shown in Table 3. The lags that had significant regression slopes in the GLM model are marked in this table. The maximum impact of turbidity on the rate of GIH events in the elderly was associated with turbidity values above the turbidity standard of 1 NTU. At a 6-day lag, turbidity >1 NTU was associated with four additional cases of GIH events per day per 100,000 elderly persons. At a 13-day lag, turbidity contributed 2.7 additional GIH events per day per 100,000 elderly persons.

**Table 3:** Generalized Additive Mode estimates of excess daily rates of gastroenteritis-related emergency room visits and hospitalizations in the elderly in south and central water supply areas during the 1993 outbreak, Milwaukee, Wisconsin^a^

Lag (d)	0.0–0.29 NTU^b^	0.3–0.49 NTU	0.5–0.99 NTU	1.0–2.0 NTU
0	0.57	0.57	0.56	0.56
1	0.40	0.50	0.72	1.20
2	0.27	0.37	0.58	1.12
3	0.37	0.46	0.64	1.10
4	0.33	0.54	0.94	1.64
**5** ^c^	0.31	0.66	1.38	**3.94**
**6** ^c^	0.22	0.59	1.33	**4.10**
**7** ^c^	0.15	0.44	1.02	**3.00**
8	0.24	0.61	1.30	1.97
9	0.29	0.54	0.97	1.79
10	0.17	0.54	1.28	2.53
11	0.18	0.45	0.95	1.97
12	0.24	0.46	0.87	2.01
**13** ^c^	0.21	0.58	1.29	**2.68**
14	0.09	0.49	1.23	2.38
15	0.22	0.48	0.97	1.80
16	0.37	0.52	0.76	1.61
17	0.34	0.50	0.78	1.05
18	0.38	0.44	0.52	1.03
Average daily excess rate and standard deviation	0.28 (0.11)	0.51 (0.17)	0.95 (0.28)	1.97 (0.96)
Total excess rate for all lags	5.34	9.73	18.11	37.48

^a^Per 100,000 persons at 0–18 days lag postexposure at different levels of drinking water turbidity. ^b^NTU, Nephelometric Turbidity Units. ^c^Lags in bold had statistically significant regression slopes (p <0.05).

On the basis of crude estimates of rates (Table 2), of 55 GIH events in the elderly recorded by the HCFA database during 28 days of the outbreak in south and central water supply areas, 39 (71%) exceeded of the pre-outbreak level. On the basis of the estimates of GIH excess rates in the elderly (Table 3), the total excess rate associated with turbidity >1 NTU at time lags from 0 through 18 days was 37.5 per 100,000. This rate translates into 30 excess cases of emergency room visits and hospitalizations in the 79,698 elderly in south and central Milwaukee associated with turbidity >1 NTU. Of these 30 excess cases, 18 cases (60%) occurred at time lags from 0 through 10 days postexposure (primary cases), and 12 cases (40%) occurred at time lags from 11 through 18 days postexposure (secondary cases).

## Discussion

Our first finding is a positive association between age and emergency room visits and hospitalizations due to acute gastroenteritis in elderly (persons >65 years of age) in Milwaukee. This association was significant by age category before the outbreak (p=0.001), and significant increases were present in each category during the epidemic (p=0.002) when compared to the pre-outbreak period. These increases are consistent with age-related susceptibility to gastrointestinal infections in the elderly. Our second finding is that statistically significant associations existed between elevated drinking water turbidity and increased rate of acute gastroenteritis in the elderly at time lags of 5, 6, 7, and 13 days during the epidemic but not in the pre-epidemic period.

**Figure 5:**
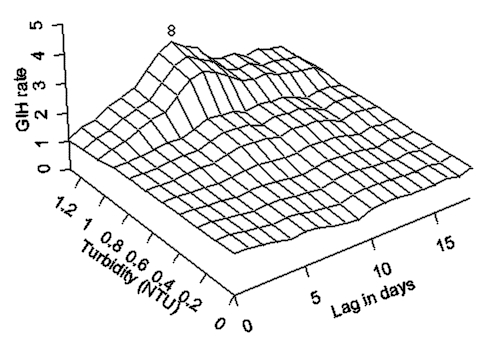
The temporal exposure response surface plot of the lagged association between daily rate of gastroenteritis-related emergency room visits and hospitalizations in all adults in the south and central water supply area of Milwaukee, Wisconsin (per 100,000) and water turbidity (Nephelometric Turbidity Units) at the south plant.

In our previous study, we argued that the characteristic time lag period between a surrogate for exposure to *Cryptosporidium* oocysts, such as turbidity, and acute gastroenteritis is indicative of the incubation period for this pathogen (*15*). Our earlier analysis of the Milwaukee outbreak demonstrated that acute gastroenteritis cases in all adults peaked at 8–9 days postexposure to contaminated drinking water. For the purposes of comparison, we have produced the TERS plot, demonstrating the relationship between daily rates of emergency room visits and hospitalizations for gastroenteritis in all adults >17 years of age and drinking water turbidity (Figure 5) by using the datasets that we analyzed in our previous publications (*13*–*15*). In the current study, we found that the first peak in the rate in GIH events in the elderly occurred at time lags of 5–6 days (Figure 4). A comparison of these plots suggests a shorter median incubation period of cryptosporidiosis in the elderly than in all adults.

A difference in incubation periods, given the same pathogen, can be due either to a different dose of the pathogen or different host susceptibility. Experiments in genetically uniform γ-interferon deficient mice have shown that the prepatent and incubation periods for *Cryptosporidium* are inversely related to the dose of parasites and can be shortened by approximately 2 days by increasing the inoculum dose by an order of magnitude (*17*,*18*). In one human volunteer study (in which the participants were not genetically uniform), an inverse (but not statistically significant) relationship was found between the dose of oocysts administered and the time to onset of infection (*20*). However, in the absence of any evidence that the dose of *Cryptosporidium* delivered through the public drinking water supply was substantially different (and certainly not an order of magnitude greater) for the elderly than for all adults in Milwaukee, we conclude that the observed difference in median incubation periods is probably due to a higher inherent host susceptibility to *Cryptosporidium* infection in the elderly.

The second peak in GIH rates associated with increased water turbidity occurred at 13 days postexposure. We believe that this second peak reflects the wave of secondary transmission of cryptosporidiosis from primary waterborne cases to the elderly. First, this hypothesis is consistent with an approximate doubling of the 7-day mean incubation period of cryptosporidiosis reported from this and other epidemics of cryptosporidiosis (*12*,*15*,*21*–*25*). Second, in human volunteer experiments (*18*,*20*) and a variety of other outbreaks of cryptosporidiosis (*19*), incubation periods of >13 days were very unusual.

Our third finding relates to the magnitude of the second peak in GIH rate in the elderly, the peak of presumed secondary transmission. Approximately 40% of excess GIH events associated with turbidity >1 NTU occurred in time lags most consistent with secondary spread. Furthermore, the relative magnitude of the increase in gastroenteritis rate at time lags consistent with secondary spread was more pronounced in the elderly than in all adults >17 years of age. (Figures 4 and 5, respectively). This finding suggests that the elderly may have a relatively higher risk for secondary person-to-person transmission. This higher risk for secondary transmission could theoretically be caused by a higher susceptibility of the elderly, a higher likelihood of exposures, especially among the elderly residing in nursing homes, or both. Relatively little is known about the overall risk for secondary transmission of *Cryptosporidium* after its introduction during an epidemic (*19*,*26*,*27*). MacKenzie et al. reported a secondary transmission rate in visitors to and residents of Milwaukee of 4.2% to 5%, but that study did not focus on the elderly (*24*). Our results in no way contradict those data.

In a retrospective microbiologic review from Rhode Island, 13 (36%) of 36 hospitalized patients identified as having had cryptosporidiosis were 63–93 years of age, with a mean of 77 years of age, and 7 of the 13 were believed to have acquired the infection nosocomially (*28*). Little other information about this disease in the elderly is available. A random-digit telephone survey of Milwaukee residents (*12*) after the 1993 outbreak identified the elderly (>70 years of age) as the subgroup with the lowest attack rate of watery diarrhea (14% vs. 26% in the general population), yet Proctor et al. (*6*) found that diarrhea was highly prevalent in the elderly in nursing homes during the outbreak. These apparently discrepant results may be the natural result of different subgroups of the elderly. In our analysis, we used age-specific data on emergency room visits and hospital admissions among all elderly as captured by the HCFA database to demonstrate that age was a risk factor among the elderly for gastroenteritis both before and during the outbreak.

While the elderly in general are more susceptible to gastrointestinal infections than other adults, gastrointestinal infections are especially prevalent in those residing in nursing homes and other similar institutions. Infectious diarrhea is the fourth most common infectious disease in the elderly residing in long-term care facilities (*7*). Studies have estimated the annual incidence of infections to be 1 to 2.59 per year in the elderly living in nursing homes (*4*,*5*,*29*) versus 0.69 per year in the elderly living in the community (*30*). Outbreaks of infectious intestinal disease are common in nursing homes and are associated with high attack rates, prolonged duration, and high disease and death rates (*29*,*31*).

Cryptosporidiosis is underdiagnosed and underreported (*32*,*33*). Cryptosporidiosis is likely to be an unrecognized cause of diarrhea in the elderly, perhaps mimicking or occurring in combination with *Clostridium difficile* (*28*), a well-known agent of diarrheal illness in nursing homes. Diagnostic testing for cryptosporidiosis was rarely performed before and during the Milwaukee epidemic. As most cases of cryptosporidiosis were likely to be misdiagnosed as either noninfectious gastroenteritis or masked by other pathogens, we used all ICD-9 codes that could potentially reflect cases of waterborne cryptosporidiosis.

Human volunteer studies have also established that many persons infected with *Cryptosporidium* are asymptomatic or mildly ill (*20*,*34*). Thus, most cryptosporidiosis cases were not reflected in the HCFA database in which relatively severe cases (seeking medical attention or hospitalization) were captured. While this database does not permit us to comment on these mildly ill persons, it does allow us to study the severely ill.

Among the elderly, publicity about the outbreak may have caused an increased concern and increased hospital visits. However, the peak in gastrointestinal hospitalizations among the elderly occurred on April 3, four days before the Milwaukee Health Department reported the outbreak and 5 days after the peak in water turbidity (Figure 3). Therefore, our results are unlikely to be biased by the publicity of the outbreak. In time-series analysis, in which participants serve as their own controls, the responses on a given day are compared with responses in the same population (or sample) on specified previous days. Thus, interpersonal confounding factors and biases that frequently affect the results of cross-sectional and longitudinal studies with different exposure and control groups did not affect the results of time-series analysis. However, other factors that vary in time and are correlated with both the exposure and the outcome may indeed confound the results of time-series data analysis. Our final statistical model included the most influential time-varying factor, the day of the week. In addition, the model included a set of autoregressive components to control for potential lack of temporal independence of observations. The analysis of model residuals demonstrated the adequacy of this model.

While the breakdown in the water treatment filtration process may have allowed other pathogens to enter the public drinking water supply, no concrete evidence has been published. Thus, we suspect that most, if not all, of the increase in gastroenteritis detected in the elderly during this period was likely due to cryptosporidiosis. In nonoutbreak situations, cryptosporidiosis accounts for 0.5% to 5% of all cases of acute gastrointestinal illness (*14*,*21*,*35*). If we assume that most of the observed gastroenteritis increase in the elderly during the epidemic was due to *Cryptosporidium* infection, then the estimated magnitude of the increase in severe cryptosporidiosis cases in the elderly that resulted in hospitalization or emergency room visits is 30- to 300-fold.

The incidence of waterborne disease has been shown to be associated with the type of water supply; it is higher in communities with unfiltered surface water or mixed unfiltered surface and ground water supplies (*36*,*37*). Drinking water contamination with pathogens, such as *Giardia* and *Cryptosporidium*, has been shown to correlate with drinking water turbidity (*38*–*40*). In recognition of the importance of turbidity as an indicator of microbiologic safety of drinking water, the U.S. Environmental Protection Agency has recently released more stringent regulations to control drinking water turbidity (see Federal Register, 66:3770 and 67:1812, the Interim Enhanced Surface Water Treatment Rule and the Long Term 1 Enhanced Surface Water Treatment Rule, respectively) (*41*,*42*). Our analysis reflects the use of finished drinking water turbidity as a surrogate variable reflecting exposure to *Cryptosporidium* oocysts in water. The data on actual concentration of *Cryptosporidium* oocysts in tap water before and during the Milwaukee outbreak are not available because no prospective *Cryptosporidium* monitoring was conducted at that time.

The analytical tools we have developed in this and our previous studies allowed us to estimate the total number of attributable cases for primary waterborne exposures and for secondary transmission. In this study, we expanded our previously developed methods by estimating the excess cases associated with increased water turbidity. Standard epidemiologic investigations usually require a history of exposure to a known primary case of disease in order to link a secondary case to the outbreak. This requirement may result in an underestimation of secondary transmission, since the persons involved in this chain of transmission may not recall or recognize their contacts. Thus, the novel statistical technique that we applied for this analysis may have broad applicability to estimating the impact of secondary infections during outbreaks.
